# Differences in the performance of health officers at the workplace according to their qualifications

**DOI:** 10.1186/s40557-018-0246-8

**Published:** 2018-05-30

**Authors:** Yeong-Kwang Kim, Jin-Ha Yoon, Wanhyung Lee, Jihyun Kim, Sung-Shil Lim, Jong-Uk Won

**Affiliations:** 1 The Institute for Occupational Health, Yonsei University College of Medicine, Seoul, Republic of Korea; 2 Department of Preventive Medicine, Yonsei University College of Medicine, Seoul, Republic of Korea; 3 Graduate School of Public Health, Yonsei University, Seoul, Republic of Korea; 4 Department of Occupational and Environmental Medicine, St. Mary’s Hospital, College of Medicine, the Catholic University of Korea, Seoul, Republic of Korea

**Keywords:** Health officer, Nurse, Industrial hygienist, Air environmental engineer

## Abstract

**Background:**

Health officers are an integral part of the occupational health service, and there have been studies to identify and improve the role of health officers in the workplace in order to improve the level of health care in the workplace. This study aimed to determine the contribution of health officers to the role of a health officer as prescribed by law and the percentage of health management work performed during work according to their qualifications.

**Methods:**

Questionnaires were distributed to a total of 4584 workplaces where health officers were hired, and a total of 806 copies (17.58%) were returned. Of these, 336 questionnaires were finally analyzed, after excluding questionnaires missing the main variables. Using the data, the difference of role contributions and the percentage of health care work performed during the whole day according to the qualification of the health officer was analyzed.

**Results:**

Nurses were highly rated in the field of medical care, and industrial hygienists and air environmental engineers were highly rated in terms of chemicals and risk factor management. The percentage of health care work performed during the whole day differed according to the size of the workplace and industrial classification, but it was generally the lowest among air environmental engineers.

**Conclusions:**

Health officers play a very different role in the workplace depending on their qualification, and they need support for areas of other qualification. In order to effectively manage the health of the staff at a workplace, it is necessary to consider the development of a support system for small- and medium-sized enterprises and adjust the conditions of employment of the health officer according to the law.

## Background

Studies have shown that the economic costs of occupational injuries and illnesses range from 1.8 to 6% depending on the country. [1] In the EU, the economic costs incurred by occupational injuries and illnesses are estimated to be 15 times the cost of preventing them. [2] Thus, prevention of occupational injuries and illnesses through occupational safety measures and health services is important.

In Korea, the number of industrial accidents has decreased with time. As of 2005, the industrial accident rate was 0.77%, and the death rate due to industrial accidents per 10,000 employees was 2.06; these rates were 0.5% and 1.01, respectively, in 2015. [3, 4] Moreover, in Korea, the incidence of fatal occupational injuries was 5.3 per 100,000 workers in 2015. However, in terms of Organisation for Economic Co-operation and Development member countries, the incidence was 1.6 per 100,000 workers in Germany and 3.3 per 100,000 workers in the USA. Efforts to improve the level of health management at the workplace are therefore needed. [5].

Health officers are an integral part of the occupational health service [6], and previous studies have aimed to identify and improve the role of health officers in the workplace in order to improve the level of health care in the workplace. In Korea, a health officer is defined as a person who assists a business owner or the person in charge of management with respect to technical matters concerning the health of the staff as well as advises and instructs the supervisor on such matters. Health officers are professionals such as doctors, nurses, industrial hygienists, and air environmental engineers [7, 8].

In Korea, the number of health officers employed in an institution varies depending on the type of industry and the number of workers in the workplace. For example, a business with a size of 500 to 2000 workers requires two or more people to be employed as health officers, and a business with a size of more than 2000 people may require the employment of two or more health officers including at least one physician or nurse by profession. [8] As such, only a limited number of industries require that at least one physician or nurse be appointed among the two or more health officers. Thus, there has been a question of whether it is possible to comprehensively perform the various roles of health officers, such as workers’ safety guidance, hazard risk factor management, and medical practices.

To address this problem, previous studies have aimed to determine the role of health officers with different qualifications. Hong et al. used a questionnaire based on the Industrial Safety and Health Law to determine the competence of health officers according to their qualifications; however, only doctors, nurses, and industrial hygienists were compared. [9] Lee et al. compared the performances and behaviours of health officers according to their qualifications, and reported a difference in the performance of nurses, industrial hygienists, and air environmental engineers. [10] However, no further studies have been conducted after a long period of time.

This study aimed to determine the contribution of health officers to health management work per the criteria prescribed by the law in Korea and the percentage of health management work performed during an actual working day according to their qualifications.

## Methods

### Subjects

A questionnaire was mailed from August to September 2015 to entire workplaces in Korea where health officers were hired. Health officers were requested to answer the questionnaire, and in case of multiple health officers, only one person answered the questionnaire. Questionnaires were distributed to a total of 4584 workplaces nationwide, and a total of 806 copies (17.58%) were returned. After excluding questionnaires missing the main variables (sex, age, size of workplace, type of business, total industrial health career, role contribution), 336 questionnaires were finally included in the analysis.

### Qualifications

At the time of the survey, the qualification of a health officer in Korea includes physicians, nurses, industrial hygienists, air environmental engineers, industrial health instructors, and those who majored in occupational health or industrial hygiene at a college. In this study, 94.7% of the workplaces surveyed were found to employ nurses, industrial hygienists or air environmental engineers as health officer. Other qualifications such as physician were excluded because the number of them was insufficient to analyze. In addition, questionnaires filled out by health officers with multiple qualifications were excluded.

### Questionnaire

The administered questionnaire was based on the Industrial Safety and Health Act. A total of 17 question items were selected based on the roles of health officers defined by the Industrial Safety and Health Law (Table 1). The questions aimed at determining the role contribution of the health officer was as follows: “As a health officer, how much do you contribute to the following items? Please indicate your contribution to the role on a scale of 0 to 10.” In this question, 0 means “No role contribution”, and 10 means “Maximum role contribution.” In this study, the high and low role contributions were divided by the median of each item in the questionnaire. The questionnaire also sought information on the percentage of health care work performed during the whole day. In addition, the following questions were asked to identify areas that need support in the field of occupational safety and health: “Which of the following areas do you need support for? - Safety and environmental management/Health management/ Administrative support.” The size of the workplace and the type of business, as well as the sex, age, and total industrial health career of the health officer were also identified.

**Table 1:** Question items based on the roles of health officers defined by the Industrial Safety and Health Law

Abbreviation	Question^a^
Occupational health and safety committee	Establishment and management of occupational health and safety committee
Safety and health actions on machinery and equipment	Matters concerning safety and health actions when harmful or dangerous machinery and equipment and other facilities are introduced
Advice and guidance on protectors	Assistance to, and advice and guidance on selection of appropriate products in purchasing protectors
Management of chemical substances	Management of chemical substances and products containing a chemical substance
Detecting harmful or dangerous factors	Detect harmful or dangerous factors caused by those resulting from specific work behaviors or duties, and determine the degree of danger
Preventing any danger or health impairment	Actions for preventing any danger to or health impairment of employees
Appropriate measures for the workers’ health	Taking appropriate measures for the workers’ health following the results, including work arrangements, work conversions, and reduction of working hours
Investigating and preventing worker’s medical problem	Investigation into the cause of workers’ medical problems and medical treatment to prevent recurrence
Health education	Assistance to, and advice and guidance on the formulation of plans on health education and the conduct thereof at the place of business concerned
Treatment of minor injuries	Treatment of frequently occurring minor injuries, such as external wounds
Emergency treatment	Emergency treatment
Preventing injuries or diseases from worsening	Treatment to prevent injuries or diseases from worsening
Management of workers after a medical checkup	Recuperation guidance and management for those who are found to have health trouble after a medical checkup
Management of ventilators and local air exhausters	Assistance to and advice on the inspection of facilities, such as general ventilators and local air exhausters, etc., and the technical improvement of working method
Routine inspections of workplace	Routine inspections of the place of business concerned, guidance and recommendation of safety measures
Investigating and preventing industrial accidents	Investigation into and analysis of the cause of industrial accidents and provision of technical assistance to and advice and guidance on prevention of the reoccurrence
Analysis of industrial accidents	Assistance to, and advice and guidance on the maintenance, management and analysis of statistics on industrial accidents

^a^The question was “As a health officer, how much do you contribute to the following items? Please indicate your contribution to the role on a scale of 0 to 10”

### Statistical analysis

All subjects were divided into groups according to their qualifications. The average and standard deviations of role contributions established by health officers themselves for each job item defined by law were obtained. The differences were analysed via one-way analyses of variance and corrected for multiple comparisons using the Bonferroni correction. The percentage of health care work performed during the whole day was analysed in a similar manner, and was stratified into manufacturing industry, non-manufacturing industry, workplace with less than 300 employees, and workplace with more than 300 employees. In addition, the differences in the percentage of areas that need support according to qualifications were illustrated.

Logistic regression analysis was used to confirm the association between the qualification of the health officer and the role contribution of the health officer. For industrial hygienists and air environmental engineers, we calculated odds to account for the assumption that role contributions would be higher for each of the items when compared with nurses. The odds ratio and 95% confidence interval were calculated by adjusting for confounding variables such as sex, age group, size of workplace, total career of industrial health, and industrial classification. Statistical analysis was performed using the SAS 9.4 (SAS Institute Inc., Cary, NC, USA.)

## Result

### General characteristics

Of the 336 subjects, nurses accounted for 67.0%. Among nurses, the percentage of women was very high (98.7%), and for other professionals, the percentage of men was higher. The proportion of health officers aged 40 to 49 years was higher among nurses than among other professionals. Workplaces with less than 300 employees accounted for the majority of those included. Nurses and industrial hygienists most frequently worked for less than 5 years, but air environmental engineers reported more than 10 years of work. By industry, other industries were the most common; however, this is typically observed in medical institutions where a nurse is working while being employed as a health officer. Other than that, manufacturing was the most common (Table 2).

**Table 2:** General characteristics of subjects stratified by qualifications of health officers

Characteristics	Nurse n (%)	Industrial hygienist n (%)	Air environmental engineer n (%)
Total	225 (66.96)	34 (10.12)	77 (22.29)
Sex			
Men	3 (1.33)	23 (67.65)	72 (93.51)
Women	224 (98.67)	11 (32.35)	5 (6.49)
Age group (years)			
20 to 29	20 (8.69)	9 (26.47)	6 (7.79)
30 to 39	54 (24.00)	11 (32.35)	32 (41.56)
40 to 49	91 (40.44)	9 (26.47)	27 (35.06)
50 and over	60 (26.67)	5 (14.71)	12 (15.58)
Size of workplace			
< 300 workers	167 (74.22)	13 (38.24)	67 (87.01)
300 to 499 workers	19 (8.44)	4 (11.76)	7 (9.09)
500 to 999 workers	20 (8.89)	8 (23.53)	1 (1.30)
above 1000 workers	19 (8.44)	9 (26.47)	2 (2.60)
Total career of industrial health			
less than 5 years	127 (56.44)	16 (47.06)	29 (37.66)
5 to 9 years	43 (19.11)	8 (23.53)	17 (22.08)
10 years and over	55 (24.44)	10 (29.41)	30 (40.26)
Industrial classification			
Manufacturing	41 (18.22)	25 (73.53)	66 (85.71)
Construction	5 (2.22)	1 (2.94)	0 (0.00)
Trasportation/Warehousing/Networking	0 (0.00)	1 (2.94)	1 (1.30)
Energy/Water supply	2 (0.89)	0 (0.00)	2 (2.60)
Financing/Insurance	1 (0.44)	0 (0.00)	2 (2.60)
Mining	1 (0.44)	0 (0.00)	0 (0.00)
Agriculture/Forestry/Fishery	1 (0.44)	0 (0.00)	0 (0.00)
Others	174 (77.33)	7 (20.59)	6 (7.79)

### Role contribution

Industrial hygienists and air environmental engineers showed higher role contribution scores than nurses in the following 8 items: “Establishment and management of occupational health and safety committee”; “Matters concerning safety and health actions when harmful or dangerous machinery and equipment and other facilities are introduced”; “Assistance with and advice and guidance on selection of appropriate products when purchasing protectors”; “Management of chemical substances and products containing a chemical substance”; “Assistance with and advice on the inspection of facilities such as general ventilators and local air exhausters and technical improvement in the working method”; “Routine inspections of the place of business concerned, guidance and recommendation of safety measures”; “Investigation into and analysis of the cause of industrial accidents and provision of technical assistance to and advice and guidance on prevention of recurrence”; “Assistance with and advice and guidance on the maintenance, management, and analysis of statistics on industrial accidents” (Table 3).

**Table 3:** The differences of role contiribution and percentage of health care work during the whole day of health officers by qualifications

The role contiribution of health managers	Nurse		Industrial hygienist		Air environmental engineer		Comparison*
	Mean	SD	Mean	SD	Mean	SD	
Occupational health and safety committee	4.95	2.79	6.24	2.51	5.86	2.56	B > A, C > A**
Safety and health actions on machinery and equipment	5.04	2.99	6.65	2.27	6.10	2.50	B > A, C > A
Advice and guidance on protectors	5.99	2.92	7.32	2.18	7.22	2.16	B > A, C > A
Management of chemical substances	6.31	2.86	7.53	1.93	7.60	1.85	B > A, C > A
Detecting harmful or dangerous factors	5.64	2.76	6.35	2.39	6.65	2.14	C > A
Preventing any danger or health impairment	6.45	2.68	7.24	2.16	6.92	2.02	
Appropriate measures for the workers’ health	6.49	2.86	6.09	2.84	6.21	2.48	
Investigating and preventing worker’s medical problem	6.55	2.82	5.68	2.59	5.56	2.77	A > C
Health education	7.22	2.47	7.29	2.17	7.18	2.12	
Treatment of minor injuries	7.46	3.00	4.35	2.96	4.10	3.05	A > B, A > C
Emergency treatment	7.68	2.77	5.43	3.18	4.57	2.97	A > B, A > C
Preventing injuries or diseases from worsening	7.55	2.79	5.27	3.14	4.45	3.07	A > B, A > C
Management of workers after a medical checkup	7.78	2.50	6.21	2.21	5.48	2.80	A > B, A > C
Management of ventilators and local air exhausters	4.78	2.89	6.29	2.42	6.24	2.46	B > A, C > A
Routine inspections of workplace	5.90	2.56	6.97	2.11	7.04	2.11	B > A, C > A
Investigating and preventing industiral accidents	5.16	2.79	6.59	2.32	6.76	2.31	B > A, C > A
Anaylsis of industrial accidents	5.05	2.90	6.53	2.45	6.35	2.34	B > A, C > A
Percentage of health care work during the whole day	Nurse		Industrial hygienist		Air environmental engineer		Comparison*
	Mean	SD	Mean	SD	Mean	SD	
Manufacturing industry	74.39	27.75	53.04	30.20	23.89	21.82	A > B, B > C
Non-manufacturing industry	32.02	31.10	75.00	28.78	21.18	14.03	B > A, B > C
The workplaces with 300 employees or less	26.70	26.68	37.92	28.40	21.22	18.84	
The workplaces with more than 300 employees	77.41	26.11	70.05	26.33	38.00	27.41	A > C, B > C

*The differences were analyzed by one-way ANOVA and corrected by multiple comparisons using bonferroni correction. *P*-value< 0.05

**“B > A, C > A” means a statistically significant higher score or percentage of industrial hygienist(B) than Nurse(A) and a statistically significant higher score or percentage of air environmental engineer(C) than Nurse(A). However, there is a no significant difference between Industrial hygienist(B) and Air environmental engineer(C)

SD, Standard deviation

Nurses showed higher role contribution scores than other professionals in the following 4 items: “Treatment of frequently occurring minor injuries, such as external wounds”; “Emergency treatment”; “Treatment to prevent injuries or diseases from worsening”; “Recuperation guidance and management for those who are found to have health trouble after a medical check-up” (Table 3).

Air environmental engineers showed higher role contribution scores than nurses for “Detection of harmful or dangerous factors resulting from specific work behaviours or duties and determination of the degree of danger.” Nurses showed higher role contribution scores than air environmental engineers for “Investigation into the cause of workers’ medical problems and medical treatment to prevent recurrence” (Table 3).

The odds of higher role contributions when compared with nurses were calculated. In the case of industrial hygienists, the odds for higher role contribution were significantly higher for the following 3 items: “Matters concerning safety and health actions when harmful or dangerous machinery and equipment and other facilities are introduced”; “Management of chemical substances and products containing a chemical substance”; “Detection of harmful or dangerous factors resulting from specific work behaviours or duties and determination of the degree of danger.” However, the odds for higher role contribution were significantly lower in the following 4 items: “Treatment of frequently occurring minor injuries, such as external wounds”; “Emergency treatment”; “Treatment to prevent injuries or diseases from worsening”; “Recuperation guidance and management for those who are found to have health trouble after a medical check-up” (Table 4).

**Table 4:** Association between the qualification of the health manager and the role contribution of the health officer

The role contribution of health manager	Industrial hygienist^a^		Air environmental engineer^a^	
	OR^b^	95% CI^b^	OR^b^	95% CI^b^
Occupational health and safety committee	1.79	0.71–4.52	2.16	0.78–5.97
Safety and health actions on machinery and equipment	3.57	1.41–9.05	2.46	0.89–6.80
Advice and guidance on protectors	2.46	0.98–6.22	2.14	0.78–5.90
Management of chemical substances	2.94	1.16–7.46	4.72	1.69–13.17
Detecting harmful or dangerous factors	2.54	1.01–6.40	2.78	1.01–7.67
Preventing any danger or health impairment	1.98	0.79–5.00	2.00	0.73–5.52
Appropriate measures for the workers’ health	0.57	0.23–1.44	0.60	0.22–1.66
Investigating and preventing worker’s medical problem	0.42	0.17–1.07	0.35	0.13–0.97
Health education	0.49	0.19–1.24	0.57	0.20–1.56
Treatment of minor injuries	0.05	0.02–0.14	0.03	0.01–0.09
Emergency treatment	0.10	0.04–0.27	0.04	0.01–0.11
Preventing injuries or diseases from worsening	0.13	0.05–0.33	0.06	0.02–0.17
Management of workers after a medical checkup	0.08	0.03–0.21	0.05	0.02–0.15
Management of ventilators and local air exhausters	2.04	0.81–5.13	2.48	0.90–6.83
Routine inspections of workplace	1.72	0.68–4.32	2.35	0.85–6.46
Investigating and preventing industiral accidents	2.15	0.85–5.41	3.52	1.27–9.72
Anaylsis of industrial accidents	1.30	0.52–3.26	1.22	0.45–3.34

^a^Odds that role contributions would be higher for each of the items compared with nurse

^b^Odds ratio and 95% CI calculated using a logistic regression model adjusted for sex, age group, size of workplace, total career of industrial health, industrial classification

OR, Odds ratio; CI, Confidence interval

In the case of air environmental engineers, the odds for higher role contribution were significantly higher for the following 2 items: “Management of chemical substances and products containing a chemical substance”; “Detection of harmful or dangerous factors resulting from specific work behaviours or duties and determination of the degree of danger.” However, the odds for higher role contribution were significantly lower for the following 5 items: “Investigation into the cause of workers’ medical problems and medical treatment to prevent recurrence”; “Treatment of frequently occurring minor injuries, such as external wounds”; “Emergency treatment”; “Treatment to prevent injuries or diseases from worsening”; “Recuperation guidance and management for those who are found to have health trouble after a medical check-up” (Table 4).

### Percentage of health care work during the whole day

The percentage of health management work performed during the whole day in manufacturing industries was highest in the order of nurses, industrial hygienists, and air environmental engineers. In non-manufacturing industries, the proportion of health management work performed during the whole day was higher for industrial hygienists than for nurses and air environmental engineers. There was no significant difference in qualifications among health officers in workplaces with 300 employees or less when stratified by workplace size. Nurses and industrial hygienists in workplaces with more than 300 employees performed a higher percentage of health care work than air environmental engineers (Table 3).

### Areas that need support in the field of occupational safety and health

**Fig. 1:**
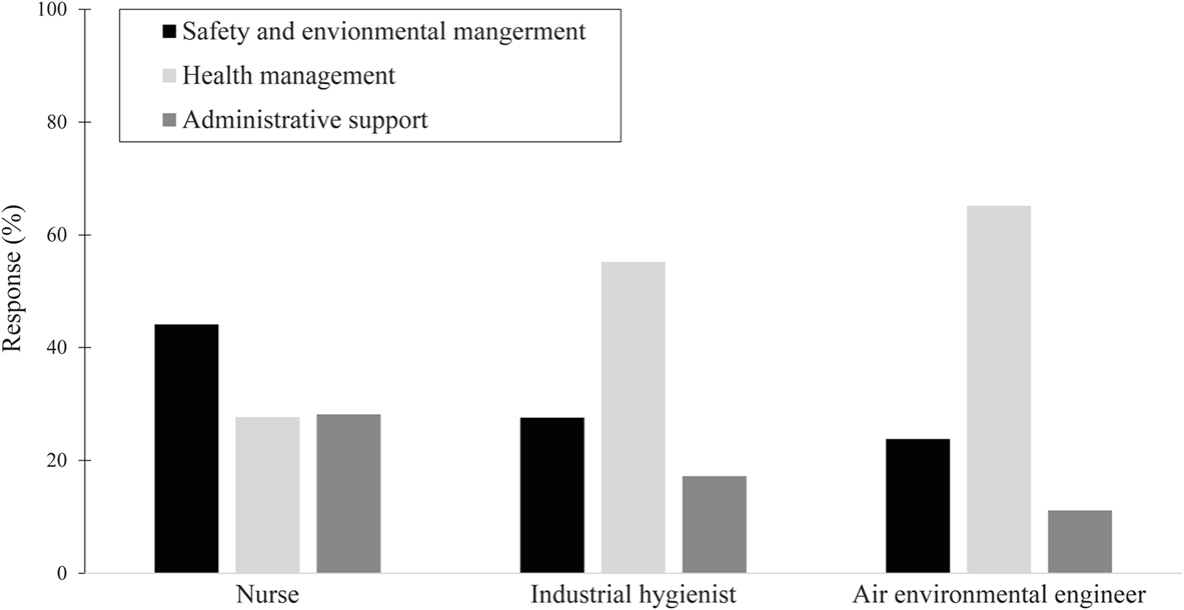
Response to question: “Which of the following areas do you need support for?”

Among the nurses, 44.1% responded that they needed support for ‘Safety and environmental management’ when they were working as health officers. There was no significant difference in the percentage of nurses who answered that they needed support for ‘health management (27.7%)’ or ‘administrative support (28.2%).’ 55.2% of industrial hygienist responded that they needed support for ‘Health management’, followed by ‘Safety and environmental management (27.6%)’ and ‘Administrative support (17.2%). In the case of the air environmental engineer, 65.1% of respondents answered that they need support for ‘ Health management’, followed by ‘Safety and environmental management (23.8%)’ and ‘Administrative support (11.1%)’ (Fig. 1).

## Discussion

In this study, there was a difference in contribution to the role of a health officer between nurses, industrial hygienists, and air environmental engineers. In the case of nurses, the contribution to items related to medical treatment was significantly higher than that observed with other professionals. On the other hand, industrial hygienists and air environmental engineers contributed more to the management and action of harmful factors.

In a study by Lee et al., nurses demonstrated a high implementation rate of health management and health planning, and industrial hygienists demonstrated a high implementation rate of work environment management, indicating a difference in contributions to the role of a health officer between professionals, similar to that observed in our study. [10] Of course, these results can be interpreted as each job category having its own strengths. However, it is more likely that the role of the health officer will not be balanced in the case of small business settings with one health officer. In addition, in large-scale workplaces hiring only a physician or nurse as a health officer may also be inadequate.

Traditionally, occupational health and safety includes occupational safety, industrial hygiene, occupational medicine, and occupational health nursing. Occupational physicians play a major role in the prevention, discovery, and treatment of occupational injuries and illness, and they may be hired or consulted at a medical institution or company. [11] In terms of clinical practice, they mainly play a role in treating workers, but when employed in a company, they often engage in administrative work as well as in medical practice. [12] Occupational health nursing is also expanding its role compared to that in the past, and these nurses act as experts, managers, or researchers of occupational health as well as medical personnel. [13] Industrial hygienists play a role in controlling potential health hazards using environmental monitoring and several other methods. [14] In Korea, however, air environmental engineers can also be appointed as health officers. In this study, although there were differences in contribution according to workplace size and type of business, the percentage of health care work performed over one entire working day was the lowest among air environmental engineers. In a study by Lee et al., 95.9% of air environmental engineers performed other tasks besides health care work. [10] Although there is no clear difference in terms of quality of health care work performed, there are the possibility of the health care at a workplace being poor if the time invested is small. As part of the deregulation of companies in Korea, an air environmental engineer hired under the Clean Air Conservation Act could also serve as a health officer, and therefore, their work may be dispersed. In our study, it was not statistically significant; however, we identify that the contribution to the role of a health officer was lowest among air environmental engineers. In addition, not only are there differences in role contribution according to the qualifications of health officers, but they responded that they need support for areas of other qualifications. Countermeasures such as reducing the additional posting of air environment engineer or strengthening education are warranted.

This study does include some limitations. This study conducted a wide range of surveys, but the response rate was low because it was conducted via mail; moreover, the responses had a large amount of missing data. There was also the disadvantage that the objectivity of the evaluation was inferior because the contributions to the role were assessed by the participants and not externally. Because of the high percentage of nurses among domestic health officers, nurses accounted for a large proportion of the respondents. Therefore, the imbalance between qualifications compared was severe, and the number of doctors was too small to conduct a comparative analysis.

## Conclusion

This study found that each qualification plays a very different role in the workplace; moreover, health officers need support for areas of other qualifications. It is difficult to effectively manage the occupational health of the workplace in Korea when only one health officer is employed at small and medium-sized workplaces. Therefore, in the case of small and medium-sized enterprises, it is necessary to consider the establishment of a support system at the local or national level for the areas where one health officer is insufficient. In addition, in the case of a workplace where two or more health officers are employed, coordination is needed to ensure that each specialty is equally deployed.

## References

[1] Takala J, Hämäläinen P, Saarela KL, Yun LY, Manickam K, Jin TW, Heng P, Tjong C, Kheng LG, Lim S (2014). Global estimates of the burden of injury and illness at work in 2012. J Occup Environ Hyg.

[2] Ahonen G. OSH and corporate competitiveness in a global context. In PEROSH Seminar at European Parliament. 2010;

[3] Ministry of Employment and Labor (2005). Statistics of industrial accidents. 2006.

[4] Ministry of Employment and Labor (2015). Statistics of industrial accidents. 2016.

[5] ILOSTAT database. International Labour Organization. http://www.ilo.org/ilostat/faces/ilostat-home/home?_adf.ctrl-state=tmypciqwp_134&_afrLoop=674516030148438#!. Accessed 17 Jan 2018.

[6] Institute of Medicine. Safe work in the 21st century: education and training needs for the next Decade's occupational safety and health personnel. Washington, DC: The National Academies Press; 2000. p. 33–5. 25077269

[7] Occupational safety and health act. Act no. 13906. Available from: http://www.law.go.kr/LSW/eng/engLsSc.do?menuId=2&query=OCCUPATIONAL%20SAFETY%20AND%20HEALTH%20ACT. Accessed 17 Jan 2018.

[8] Enforcement decree of the occupational safety and health act. Act no. 27767. Available from: http://www.law.go.kr/LSW/eng/engLsSc.do?menuId=1&query=ENFORCEMENT+DECREE+OF+THE+OCCUPATIONAL+SAFETY+AND+HEALTH+ACT&x=0&y=0. Accessed 17 Jan 2018.

[9] Hong YC, Ha EH, Jun KJ, rho YM, park HS, jo HS, lee HJ, Yang MR. job performance of occupational health personnel. Annals of Occupational and Environmental Medicine. 1997;9(3):496–507.

[10] Lee J-H, Kim K-S, Ahn Y-s (1995). A study on the practical behavior of health care managers at the worksite. Annals of Occupational and Environmental Medicine.

[11] LaDou J, Harrison R. Current occupational & environmental medicine: McGraw-Hill New York; 2007. P. 112–120.

[12] Harber P, Rose S, Bontemps J, Saechao K, Liu Y, Elashoff D, Wu S (2010). Occupational medicine practice: activities and skills of a national sample. J Occup Environ Med.

[13] World Health Organization. The role of the occupational health nurse in workplace health management. 2001.

[14] Plog BA, Niland J, Quinlan P. Fundamentals of industrial hygiene: National Safety Council Press; 2002. P. 3–31.

